# Biomarker Responses of Spanish Moss *Tillandsia usneoides* to Atmospheric Hg and Hormesis in This Species

**DOI:** 10.3389/fpls.2021.625799

**Published:** 2021-01-28

**Authors:** Xingyue Sun, Peng Li, Guiling Zheng

**Affiliations:** School of Resources and Environment, Qingdao Agricultural University, Qingdao, China

**Keywords:** atmospheric pollution, biomonitoring, hormesis, hg, oxidative stress

## Abstract

Hg is an environmental pollutant with severe biotoxicity. Epiphytic *Tillandsia* species, especially Spanish moss *T. usneoides*, are widely used as the bioindicator of Hg pollution. However, the effects of different Hg concentrations on *Tillandsia* have been rarely studied and the occurrence of hormesis in *Tillandsia* species has not been determined. In this study, *T. usneoides* was subjected to stress induced by 15 concentrations of gaseous Hg ranging from 0 to 1.8 μg m^–3^ through a misting system and then Hg content and eight common biomarkers in leaves were measured. The results showed that leaf Hg content significantly increased with Hg concentration, showing a linear relationship. However, there were no obvious mortality symptoms, indicating that *T. usneoides* showed strong resistance to Hg. Conversely, there were no simple linear relationships between changes in various biomarkers following Hg treatment of *T. usneoides* and Hg concentration. With increasing Hg concentration, malondialdehyde (MDA) content did not change significantly, superoxide anion radical content decreased gradually, superoxide dismutase (SOD) content decreased to the bottom and then bounced back, electrical conductivity increased, and glutathione (GSH) and metallothionein (MT) content increased to the peak and then dropped. The coefficient of determination of the dose-effect curves between SOD, GSH, and MT contents and Hg concentration was high, and the dose-effect relationship varied with hormesis. The present study is first to confirm hormesis induced by heavy metal pollution in *Tillandsia* species.

## Introduction

Hormesis is the phenomenon in which exposure to a low toxin dose produces stimulatory effects but that to a high toxin dose produces inhibitory effects on growth in the same organism (Calabrese and Blain, 2009). Since the coining of its concept, hormesis has been applied to plants and animals exposed to toxins, and it has been increasingly used in toxicology, risk assessment, and even agricultural production (Agathokleous et al., 2018; Calabrese, 2018). In plants, biomass, length, area, or volume of the whole plant or a part of its structure as well as different enzyme activities, pigment content, and oxidative stress parameters are used as indicators of hormesis (Carvalho et al., 2020).

*Tillandsia* spp., also known as air plants, are native to Central and South America, with over 500 known species. These species are of high ornamental value and easy for cultivation without soil, and now they are widely introduced and cultivated worldwide (Benzing, 2000). As specialist epiphytes, *Tillandsia* species can absorb moisture and nutrients from the air through their leaves. Thus, their leaves exhibit a strong absorption capacity for many atmospheric pollutants, particularly heavy metals. Many studies have shown that *Tillandsia* species are effective indicators for monitoring atmospheric heavy metals based on the content of pollutants accumulated in their leaves (Wannaz et al., 2006; Vianna et al., 2011; Schreck et al., 2016, 2020; Sánchez-Chardi, 2016; Li et al., 2019).

Furthermore, *Tillandsia* species exhibit various morphological and physiological responses to atmospheric heavy metal stress. In a previous study, *T. capillaris* was treated with different concentrations of Ni, Cu, Zn, and Pb solutions ranging from 0.5 to 10 mM for 45 min, malondialdehyde (MDA) content significantly increased following Pb treatment but did not significantly change following Ni, Cu, and Zn treatments (Wannaz et al., 2011). Similarly, there was no significant change in MDA content following the treatment of *T. albida* with 10 μM Cd^2+^ for 2 months (Kováčik et al., 2012). Following treatment of four *Tillandsia* species with 2 μM Cd^2+^ for a month, their morphology was not significantly altered, and glutathione (GSH) content did not increase significantly, although reactive oxygen species (ROS) and peroxidase (POD) contents increased significantly (Kováčik et al., 2014). Various physiological parameters were measured after transplanting *T. capillaris*, *T. recurvata*, and *T. tricholepis* in urban, agricultural, and industrial areas for 3 months, and MDA content, foliar damage index (FDI), electrical conductivity, relative water content, superoxide dismutase (SOD) and catalase (CAT) activities, total phenolics, and soluble protein content were altered in different species (Bermudez and Pignata, 2011). In other words, physiological and ecological responses greatly vary across different *Tillandsia* species following treatment with different heavy metals. Therefore, certain parameters can serve as biomarkers, but the occurrence of hormesis in *Tillandsia* species has not been determined.

Hg is an environmental pollutant with severe biotoxicity. It can spread all over the world through air, leading to severe consequences following biological enrichment and food chain amplification (Natasha et al., 2020). Studies on air pollution monitoring using *Tillandsia* species date back to the era when *T. usneoides*, known as Spanish moss, was identified to rapidly and effectively absorb Hg (Calasans and Malm, 1997). Following this, *T. usneoides* was increasingly used as a bioindicator because Hg content accumulated in its body shows a linear relationship with Hg concentration (Amado Filho et al., 2002; Bastos et al., 2004; Fonseca et al., 2007; Sutton et al., 2014). However, the effects of different Hg concentrations on *T. usneoides* have been rarely studied. Therefore, in the present study, *T. usneoides* was subjected to stress induced by different concentrations (from low to high) of gaseous Hg through a misting system. Hg accumulation and eight common biomarkers in leaves were measured to generate dose-effect curves and to explore the occurrence of hormesis in *T. usneoides*.

## Materials and Methods

### Hg-Induced Stress

**FIGURE 1:**
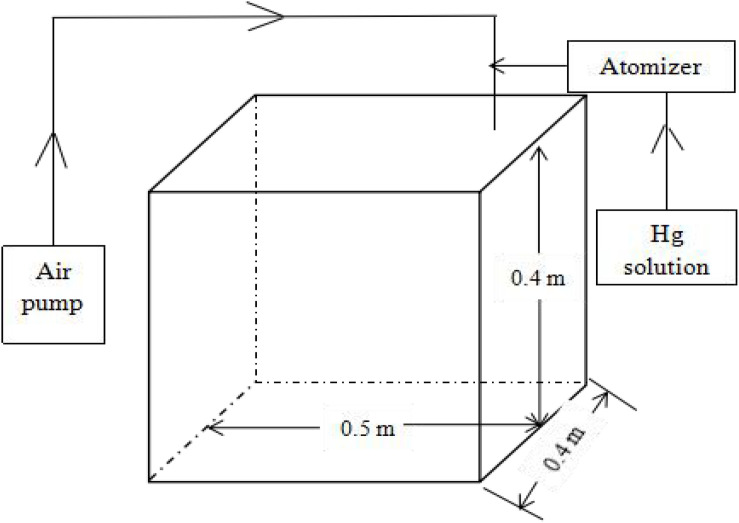
Atomizing tank used in the experiment.

Experiments were conducted in an airtight exposure box made of polymethyl methacrylate (PMMA) materials (size, 0.08 m^3^) (Figure 1). The exposure box was connected to an air pump and atomizer, and the air pump was powered continuously to generate the misting system. The atomizer misted a standard Hg solution from an external syringe to generate an aerosol misting environment. A fan was running in the middle of the box to evenly mix Hg.

The average Hg reference limit per year is 0.05 μg m^–3^ (Ministry of Environmental Protection, China, 2012), so the following 15 Hg concentrations were set (from low to high): 0, 0.02, 0.04, 0.06, 0.08, 0.1, 0.2, 0.4, 0.6, 0.8, 1, 1.2, 1.4, 1.6, and 1.8 μg m^–3^. Without the real-time Hg analyzer, Hg concentrations after misting were monitored by calculation based on the following formula. In the present experiment, the volume of the Hg standard solution was set to 30 mL, the injection speed was 15 ml h^–1^, and the injection time was 2 h. The control group was treated with deionized water instead of Hg.

Concentration of Hg (μg⋅m^–3^) = Injection speed (mL⋅h^–1^) ^∗^ Injection time (h) ^∗^ Initial concentration of standard Hg solution (μg⋅mL^–1^)/Exposure box size (0.08 m^3^).

Healthy *T. usneoides* samples from the greenhouse situated in Qingdao Agricultural University were selected as materials. First, the samples were thoroughly washed with deionized water and soaked for 30 min until saturated. They were air-dried at room temperature for 30 min. *T. usneoides* samples (fresh weight 40 g) were accurately weighed and randomly divided into five groups (five replicates). Samples in each group (8 g) were suspended on a string inside the box. After sealing the atomizing tank, fumigation was performed for 6 h. Since *Tillandsia* species are Crassulacean acid-metabolizing plants and their stomata are open at night (Benzing, 2000), all experiments were conducted at night (18:00–24:00) and the room temperature was between 23 and 25°C. The samples were collected and immediately used for the determination of different parameters.

### Determination of Hg Content in *Tillandsia usneoides*

Leaves of *T. usneoides* treated with Hg were accurately weighed (fresh weight 2.0 g), placed in a thermostatic dryer at 45°C, and pulverized. Next, 0.2 g (dry weight) of samples was transferred into a 25 mL colorimetric tube and 8 mL of nitric acid and 2 mL of perchloric acid were added. The colorimetric tubes containing the samples were shaken, covered with a plastic wrap, and incubated overnight. The next day, the samples were heat digested at 100°C in a thermostatic water bath in a fume hood. The samples were shaken every 30 min until becoming clear and cooled to room temperature. The samples in colorimetric tubes were brought up to 25 mL with deionized water and shaken well for subsequent measurements. Three blank solutions were digested following the same procedure. Finally, Hg content of the samples was determined using an inductively coupled plasma atomic emission spectroscope (ICP-OES, AFS-933, Jitian Instruments, Beijing, China).

### Effects of Hg on *Tillandsia usneoides* Growth

Following treatment with different Hg concentrations, *T. usneoides* samples were immediately collected, and leaf morphology, color, and surface scales were observed and photographed. If all the leaves of *T. usneoides* turned yellow and withered, and the cell morphology inside the leaf changed evidently, it was considered death.

The cell morphology on the leaf surface and in the leaf was observed *via* scanning electron microscopy (SEM). Mature *T. usneoides* leaves were collected and fixed in glutaraldehyde for 48 h, followed by washing with distilled water. Next, the leaves were dehydrated in 30, 50, 70, 80, 90, and 100% (twice) v/v ethanol, in that order, for 10 min. The dehydrated samples were placed on glass slides and air-dried in a sealed container. The samples were observed *via* SEM (Nova nano 450, FEI, United States) after being coated using an ion sputter.

### Measurement of Biomarkers in Hg-Treated *Tillandsia usneoides*

#### Measurement of MDA Content and SOD Activity and Electrical Conductivity

According to the methods of Li (2000), relative electrical conductivity was measured using a conductometer (DDS-307A), SOD activity was measured using nitro blue tetrazolium, and MDA content was measured using TBARs (thiobarbituric acid reactive substances). Thiobarbituric acid (TBA) reacts with MDA (final product of lipoperoxidation) and other aldehyde reactive substances (mostly soluble sugars) to give a pink compound with maximum absorbance at 532 nm. In order to eliminate the influence of other aldehyde reactive substances, the optical density (OD) values were monitored at 600 and 532 nm, respectively, before analyzing the MDA contents based on its extinction coefficient according to the method of Li (2000).

#### Measurement of Superoxide Anion Radical (O_2_^–^) Content

O_2_^–^ content was measured according to the method of Lei et al. (2006). Briefly, 1 g *T. usneoides* samples were ground in 65 mol L^–1^ phosphate buffer (pH 7.8) with quartz sand using a mortar on ice. The ground samples were transferred to centrifuge tubes, and the mortar was rinsed with phosphate buffer three times. Finally, the samples were brought up to a volume of 10 mL. After filtering, the samples were centrifuged at 10,000 rpm for 10 min, and the supernatant was collected as the extract for further experiments. Next, 2 mL of the extract (1.5 mL of phosphate buffer and 0.5 mL of hydroxylamine hydrochloride) was added to each of the three tubes and incubated in a thermostatic water bath at 25°C for 20 min after mixing. Further, 2 mL of the reaction solution aspirated from each of those tubes was added to three individual new tubes containing 2 mL of 17 mmol L^–1^ *p*-aminobenzene sulfonic acid and 2 mL of 7 mmol L^–1^ α-naphthylamine. Next, the samples were incubated in a thermostatic water bath at 30°C for 30 min to react. Finally, absorbance was measured at 530 nm to calculate O_2_^–^ content.

#### Determination of GSH Content

Glutathione content was measured according to the method of Qian et al. (2013). Briefly, 0.5 g of samples was weighed and ground in 5 mL of 5% trichloroacetic acid, and the supernatant was collected after centrifuging at 1,500 rpm for 10 min. Next, 1 mL of distilled water, 1 mL of 0.1 mol L^–1^ phosphate buffer, and 0.5 mL of 4 mmol L^–1^ DTNB solution were added in a new tube and mixed. Two additional tubes were prepared. First, 1 mL of the supernatant and 1 mL of 0.1 mol L^–1^ phosphate buffer (pH 7.7) were added in each tube. Thereafter, 0.5 mL of 4 mmol L^–1^ DTNB solution was added to one tube, and 0.5 mL of 0.1 mol L^–1^ phosphate buffer (pH 6.8) was added to the other tube. The two tubes were incubated at 25°C for 10 min. Absorbance of the chromo-developing solution was measured immediately at 412 nm. Absorbance values of the mixtures in the sample tube (ODs) and blank control tube (ODc) were recorded. GSH content was calculated according to the difference in absorbance values (μmol⋅g^–1^ FW).

#### Determination of Metallothionein Content

Metallothionein (MT) content was determined using the metal-binding method. Briefly, 1.0 g of fresh plant samples was weighed and placed in a mortar and then 6 mL of precooled 0.1 mol L^–1^ Tris–HCl buffer (pH 8.6) was added. The samples were ground in an ice bath until they were homogenized and extracted overnight in a refrigerator. The supernatant was collected after centrifuging at 10,000 rpm for 10 min at 4°C. The extract was heated in a water bath at 90°C for 3 min and cooled down to room temperature. The supernatant was centrifuged at 10,000 rpm for 10 min, and the resultant supernatant was collected again. Three-times volume of precooled anhydrous ethanol was added to precipitate overnight at −20°C. The samples were then centrifuged at 10,000 rpm for 10 min. Next, 5 mL of 0.1 mol L^–1^ Tris–HCl buffer was added to dissolve the precipitate for several hours. The supernatant was collected after centrifuging at 10,000 rpm for 10 min. As one mole of MT can bind seven moles of Hg^2+^, there is 4.985 mg MT per mg Hg^2+^. Accordingly, MT content was calculated after determining Hg content of the supernatant using the method of atomic fluorescence.

### Statistical Analysis

Statistical analyses were conducted using SPSS 23.0, and one-way analysis of variance (ANOVA) was performed to determine significant differences among different Hg concentrations. Microsoft Excel was used to create figures. Dose-effect curves were generated based on scatter graphs, and the appropriate trendline was selected according to the coefficient of determination (*R*^2^), and the corresponding fitting curve was tested based on the *P* value using SPSS 23.0.

## Results

### Hg Content in *Tillandsia usneoides*

**FIGURE 2:**
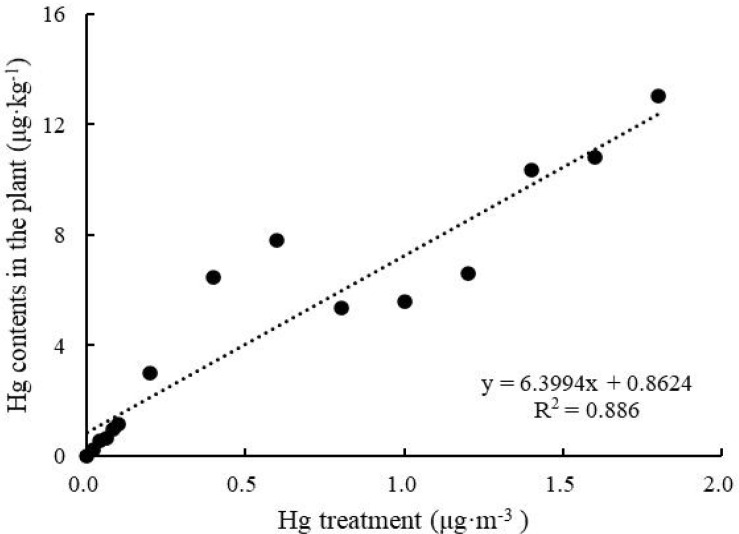
Hg contents in the plants of *Tillandsia usneoides* after treatment with different Hg concentrations.

After 6 h of treatment, leaf Hg content increased with increasing Hg concentration (Figure 2), and Hg absorption significantly increased from 0 to 13.02 ± 2.04 μg kg^–1^ (One-way ANOVA, *F* = 683.934, *P* < 0.05). The fitted curve of Hg accumulation in and Hg content of *T. usneoides* leaves was close to a line (*R*^2^ = 0.886, *P* < 0.05).

### Effects of Hg on *Tillandsia usneoides* Growth

**FIGURE 3:**
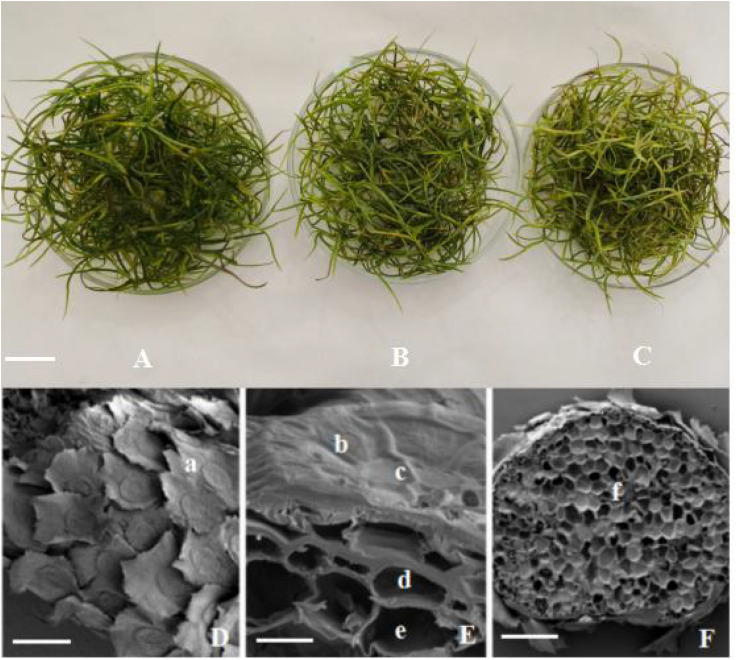
*Tillandsia usneoides* and its structure with SEM. **(A)** Before Hg stress. **(B)** After 1.0 μg m^–3^ Hg stress. **(C)** After 1.8 μg m^–3^ Hg stress. **(D)** Leaf surface before Hg stress. **(E)** Part transverse section of the leaf before Hg stress. **(F)** Whole transverse section of the leaf after 1.8 μg m^–3^ Hg stress. **(a)** Wing cell. **(b)** ring cell. **(c)** disk cell. **(d)** epidermal cell. **(e)** mesophyll cell. **(f)** vascular bundles. **(A–C)**, bar = 2 cm. **(D)**, bar = 400 μm. **(E)**, bar = 30 μm. **(F)**, bar = 400 μm.

*Tillandsia usneoides* samples treated with 0–0.8 μg m^–3^ Hg remained green (Figure 3A). The leaves turned yellow when Hg concentration increased to 1.0 μg m^–3^ (Figure 3B), and the yellowed area increased with increasing Hg concentration (Figure 3C). However, no plants (including those treated with the highest Hg concentration) exhibited marked mortality symptoms.

Scanning electron microscopy results showed that the leaf surface was covered with scales (Figure 3D), but no obvious stomatal structure was observed. The scales were sunflower-like, with long wing cells at the outermost layer, and serrated at the edges. Ring cells were observed in the middle, and disk cells were observed at the center (Figure 3E). In longitudinal section of the leaf, epidermal and mesophyll cells were laid below the scales (Figure 3E), and there were several vascular bundles among the mesophyll cells (Figure 3F). No significant differences of leaf structure were found between Hg-treated and control *T. usneoides* leaves.

### Changes in Biomarkers in Hg-Treated *Tillandsia usneoides*

#### Relative Electrical Conductivity

**FIGURE 4:**
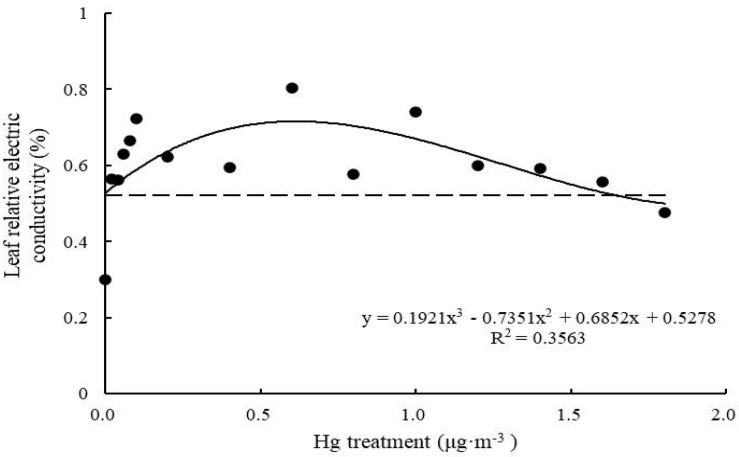
Effects of Hg on the leaf relative electric conductivity in *Tillandsia usneoides*.

Relative electrical conductivity of control *T. usneoides* leaves was 0.30 ± 0.06%. Following Hg treatment, relative electrical conductivity increased and then decreased (Figure 4). Relative electrical conductivity reached the highest value (0.8 ± 0.05%) when Hg concentration was 0.6 μg m^–3^ and the lowest value (0.48 ± 0.09%) when Hg concentration was 1.8 μg m^–3^. There were significant differences in relative electrical conductivity among different treatments (*F* = 16.75, *P* < 0.001). The fitted curve was close to an inverted U shape. Thus, there was a maximum. When the working concentration was higher or lower than the maximum concentration, the performance declined, and the curve fitting degree was not high (*R*^2^ = 0.3563, *P* < 0.05).

#### MDA Content

**FIGURE 5:**
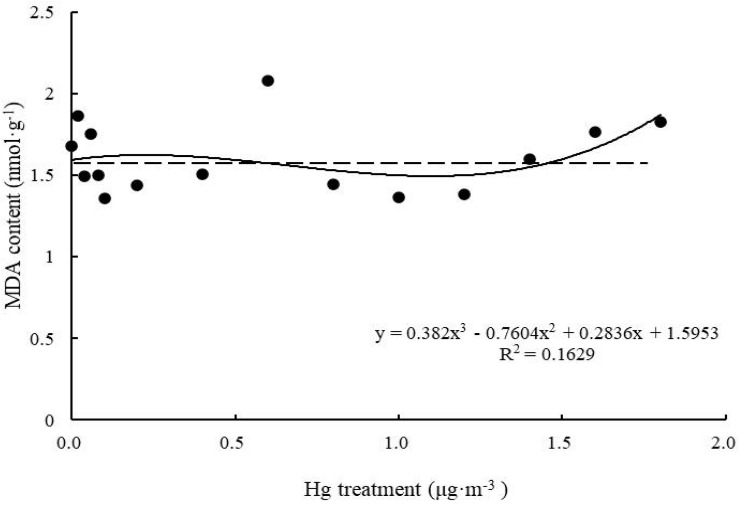
Effects of Hg on the MDA content in *Tillandsia usneoides*.

As shown in the fitted curve (Figure 5), when Hg concentration was low, MDA content of *T. usneoides* leaves spiked up, then went down, but did not fluctuate significantly (*P* > 0.05). When Hg concentration reached 1.6 μg m^–3^, MDA content increased significantly (*P* < 0.05), but the fitting degree of the curve remained poor (*R*^2^ = 0.1629, *P* > 0.05).

#### O_2_^–^ Content

**FIGURE 6:**
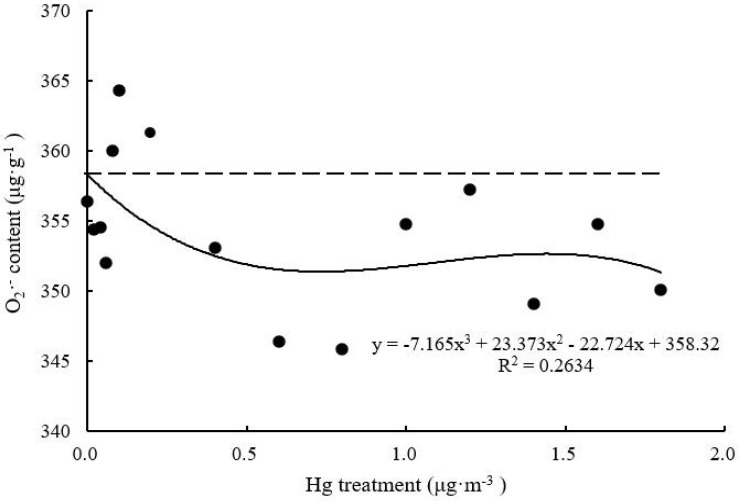
Effects of Hg on the O_2_^–^ content in *Tillandsia usneoides*.

With increasing Hg concentration, O_2_^–^ content of *T. usneoides* leaves gradually decreased and tended to be stable (*F* = 2.304, *P* < 0.05; Figure 6). The lowest O_2_^–^ content was 345.90 ± 10.95 μg g^–1^ when Hg concentration was 0.8 μg m^–3^. The R^2^ value of the fitted curve remained low (*P* > 0.05).

#### SOD Activity

**FIGURE 7:**
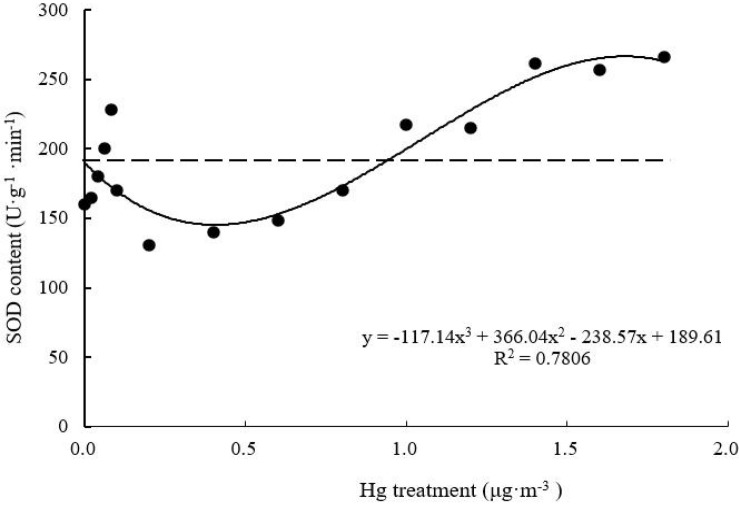
Effects of Hg on the SOD activity in *Tillandsia usneoides*.

Superoxide dismutase activity of *T. usneoides* leaves decreased and then increased with increasing Hg concentration (Figure 7). Following treatment with 0.2–0.4 μg m^–3^ Hg, SOD activity of *T. usneoides* leaves reached the lowest level. SOD activity reached the highest level at the maximal Hg concentration (265.71 ± 7.82 U g^–1^ min^–1^), and the difference in SOD activity among different Hg treatments was highly significant (*F* = 28.755, *P* < 0.001). The curve was close to a U shape, with a good degree of fit (*R*^2^ = 0.7806, *P* < 0.05).

#### GSH Content

**FIGURE 8:**
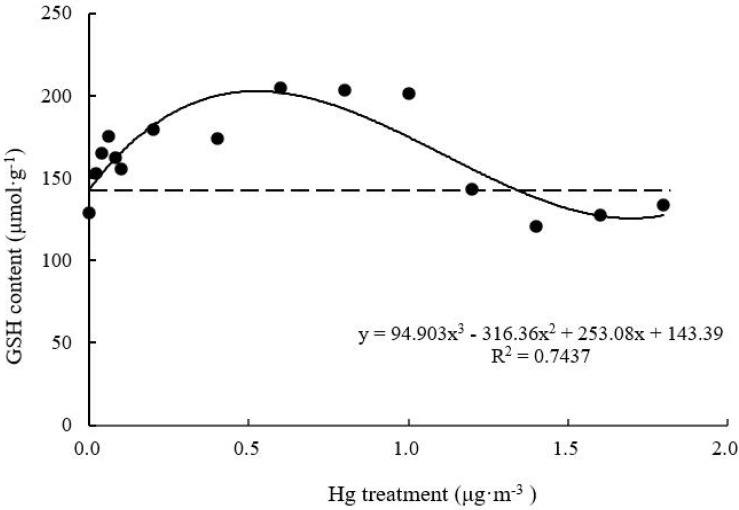
Effects of Hg on the GSH content in *Tillandsia usneoides*.

With increasing Hg concentration, GSH content of *T. usneoides* leaves increased and then decreased (Figure 8). The maximum value was 204.84 ± 8.80 μmol g^–1^ when Hg concentration was 0.6–1.0 μg m^–3^, and this value was increased by 59.0% compared with the control value. GSH content then decreased gradually. GSH content of the Hg-treated leaves was lower than that of the control leaves when Hg concentration increased to 1.4 μg m^–3^. There were highly significant differences in GSH content among different Hg treatments (factor ANOVA, *F* = 15.777, *P* < 0.001). The curve was close to an inverted U shape. Thus, there was a maximum. When the working concentration was higher or lower than the maximum concentration, the performance declined, but the curve fitting degree remained relatively high (*R*^2^ = 0.7437, *P* < 0.05).

#### MT Content

**FIGURE 9:**
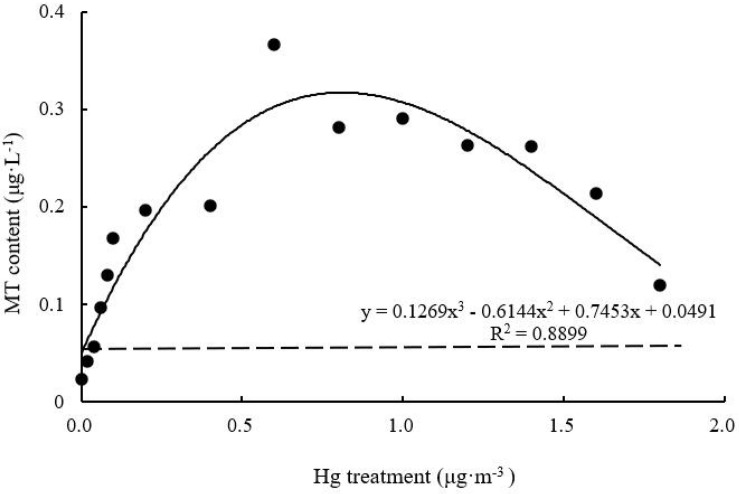
Effects of Hg on the MT content in *Tillandsia usneoides*.

With increasing Hg concentration, MT content of *T. usneoides* leaves increased and then decreased (Figure 9). MT content of Hg-treated leaves was higher than that of control leaves. The maximum content was 0.37 ± 0.1 μg L^–1^ when Hg concentration was 0.6 μg m^–3^, and this value was 18.5 times higher than the control value. MT content under different Hg treatments was significantly different (*F* = 7.496, *P* < 0.001). The curve was close to an inverted U shape, with a relatively high degree of fit (*R*^2^ = 0.8899, *P* < 0.05).

## Discussion

In the present study, *T. usneoides* was subjected to heavy metal stress induced by 15 different Hg concentrations. Given that the atomizing tank (Figure 1) may absorb some Hg, the effective Hg concentration was lower than the actual Hg concentration. There were no obvious mortality symptoms (Figure 3) when *T. usneoides* was treated with a maximum Hg concentration of 1.8 μg m^–3^, which is much higher than the atmospheric Hg concentration limit of 0.05 μg m^–3^. This result indicates that *T. usneoides* is highly resistant to atmospheric Hg pollution. This observation was consistent with the previous reports in which the *T. usneoides* could grow normally following exposure to various Hg concentrations for 15 days (Calasans and Malm, 1997; Sutton et al., 2014). In addition, in this study, with increasing Hg concentration, Hg content of *T. usneoides* leaves also increased linearly (Figure 2), suggesting that *T. usneoides* rapidly and efficiently accumulated atmospheric Hg. These results are also consistent with the previous reports (Bastos et al., 2004; Fonseca et al., 2007; Sutton et al., 2014). These observations also support the rationale that pollutant accumulation in *T. usneoides* can reflect atmospheric pollutant concentration and thus explain the reason *T. usneoides* has become a widely used bioindicator.

However, there was no simple linear relationship between changes in various biomarkers of Hg-treated *T. usneoides* and Hg concentrations. Electrical conductivity and MDA and O_2_^–^ contents reflect the damage caused by heavy metal stress (Natasha et al., 2020). However, with increasing Hg concentration, MDA content of *T. usneoides* leaves showed insignificant changes (Figure 5), while O_2_^–^ content decreased gradually (Figure 6). Only electrical conductivity showed an upward trend (Figure 4). Therefore, these results indicate that *T. usneoides* may show high resistance to Hg stress, leading to insignificant changes in parameters such as MDA and O_2_^–^ contents. Meanwhile, these results also show that among the three parameters tested, electrical conductivity is a more sensitive indicator of damage caused by Hg stress.

Glutathione, MT contents, and SOD activity reflect the plant’s ability to effectively mitigate heavy metal damage (Natasha et al., 2020). However, these parameters varied with varying Hg concentration (from low to high) in this study. SOD activity decreased and then increased (Figure 7), while GSH and MT contents increased and then decreased (Figures 8, 9). These trends indicate that GSH and MT production is significantly mediated under stress induced by low Hg concentration; therefore, these parameters are more sensitive than SOD in terms of response to Hg stress. However, with increasing Hg concentration, GSH and MT contents tended to decrease, while SOD activity increased simultaneously (Figures 7–9). Therefore, these factors for the mitigation of heavy metal damage may coordinate with one another and play different roles at different stages.

Among all parameters tested, only the dose-effect curve between SOD, GSH, and MT contents and Hg concentration showed a high degree of fit, assuming the shape of a hormetic curve (a U or an inverted U shape) (Mushak, 2013; Agathokleous et al., 2018). As a precursor of phytochelatins, GSH is a metal chelating agent with a high affinity toward metal ions (Jozefczak et al., 2012). The unique cysteine structure of MT also shows high affinity toward metal ions. Sinaei et al. (2018) found that MT shows high affinity toward Cd, Cu, Zn, Pb, Ni, Hg, and Cr in brown algae. In this study, with increasing Hg concentration, GSH and MT contents of *T. usneoides* leaves increased and then decreased, indicating that low Hg concentration induced GSH and MT production, which could greatly mitigate the damage caused by Hg and promote the growth of *T. usneoides*, resulting in hormesis. The presence of substances that can effectively alleviate heavy metal damage has been considered an important mechanism for the occurrence of hormesis (Calabrese and Blain, 2009). MT gene expression in ferns (*Azolla filiculoides*) also showed a similar variation trend under stress induced by different concentrations of Ni (Majid et al., 2019).

Typically, heavy metal treatment can improve SOD activity in plants because SOD can detoxify O_2_^–^ (ROS) (Malecka et al., 2014). In *Billbergia zebrina*, *in vitro* SOD activity increased over time and with increasing Cu concentration (Martins et al., 2016). However, Bai et al. (2014) found that SOD activity in both stem and roots of ryegrass decreased following Cd treatment. Similar to the latter report, low Hg concentration did not significantly increase SOD activity in the present study (Figure 7). These results indicate that low Hg concentration did not cause significant damage to *T. usneoides*, possibly due to hormesis as a result of GSH and MT production. When Hg concentration exceeded 1.0 μg m^–3^, GSH and MT contents declined, but SOD activity increased rapidly (Figure 7). This may be because high SOD activity can replace GSH and MT to some extent and alleviate the damage caused by Hg. However, when Hg concentration increased to 1.6 μg m^–3^, SOD activity was close to the peak; thus, its detoxification effect would not be further enhanced, resulting in significantly increased MDA content (Figure 4). These results indicate that high Hg concentration produced an expected inhibitory effect on *T. usneoides*.

In conclusion, SOD, GSH, and MT can be used as biomarkers and indicators of hormesis in response to Hg stress to some extent in *T. usneoides*. To the best of our knowledge, the present study is the first to report the occurrence of hormesis in *Tillandsia* species, which are widely used as bioindicators of atmospheric heavy metal pollution.

## Data Availability Statement

The original contributions presented in the study are included in the article/supplementary material, further inquiries can be directed to the corresponding author/s.

## Author Contributions

XS performed the experiments and analyzed the results. PL analyzed the results. GZ designed the experiments and analyzed the results. All authors contributed to the article and approved the submitted version.

## Conflict of Interest

The authors declare that the research was conducted in the absence of any commercial or financial relationships that could be construed as a potential conflict of interest.
